# Preoperative 3D and 4D-CT imaging using 640-Multislice CT (640-MSCT) in diagnosis of female urethral diverticulum

**DOI:** 10.1007/s00345-016-1965-5

**Published:** 2016-11-01

**Authors:** Yong-Xian Zhao, Jia-Ping Wang, Jiong-Ming Li, Tao Chen, Cong-Wen Mao, Jian-He Liu, Yun-Shan Su, Ke-Wei Fang

**Affiliations:** 1 Department of Urology, 2nd Hospital of Kunming Medical University, No.374, Dianmian DadaoRoad, Kunming, Yunnan 650101 China; 2 Department of Radiology, 2nd Hospital of Kunming Medical University, Kunming, Yunnan 650101 China; 3 Department of Radiology, 4th Hospital of Kunming Medical University, Kunming, Yunnan 650034 China

**Keywords:** Urethral diverticulum (UD), 640-Multislice CT (640-MSCT), 3D image, 4D image

## Abstract

**Objective:**

To determine the sensitivity and specificity of 640-Multislice CT (640-MSCT) in diagnosing the female UD.

**Materials and methods:**

We investigated 16 patients with symptomatic UDs preoperatively in our hospital from August 2010 to March 2016. The patients’ average age was 38.8 years. All patients were performed 640-MSCT of pelvis; then, 3D and 4D images were reconstructed preoperatively.

**Results:**

In 3D and 4D-CT images, out of 16 patients, thirteen patients had one ostium, two had 2 ostia and one had 3 ostia. Out of those thirteen patients, eight patients’ ostia were located at 5 o’clock and five patients’ at 7 o’clock. Patients with 2 ostia location were at 5 and 6 o’clock and 5 and 7 o’clock, respectively. Patients with 3 ostia location were at 5, 6 and 7 o’clock. The mean distance from the bladder neck to the ostia was 22.5 mm. The shape of UD was out-pouching in 11 patients (68.8%), U-shaped in four patients (25.0%) and circumferential in 1 patient (6.2%). The CT findings were confirmed by surgical findings.

**Conclusions:**

640-MSCT is a useful tool in identifying UD’s shape and ostium (including number, location) before operation. Preoperative 640-MSCT should be an adaptable modality for clinically suspected UD patients.

**Advances in knowledge:**

Several imaging methods have been used to diagnose female UD. 640-MSCT may be more suitable to diagnose it for its higher sensitivity and specificity in diagnosis of female UD, especially in identifying UD’s shape and number and location of ostium.

## Introduction

Female urethral diverticulum (UD) is affecting 0.6–6% the population with the highest prevalence occurring in their third to fifth decade [1, 2]. The most symptoms is presenting as dysuria, post-void dribbling, dyspareunia, recurrent urinary tract infections (UTIs) and swelling in anterior vagina or an anterior vaginal wall cyst [3].

It is often delayed to diagnose UD due to the fact that most of patients presenting such nonspecific genitourinary symptoms as dysuria, dyspareunia, recurrent UTIs, and so on. It is a challenging clinical problem to diagnose UD promptly and accurately. Traditionally, some UDs can be diagnosed correctly by physical examination and clinical symptoms. However, it is a pity that diagnosis of most cases depends on ultrasonography (US), multidetector computed tomography (CT) and magnetic resonance imaging (MRI), or invasive techniques such as cystourethroscopy, voiding cystourethrogram (VCUG) and urethrography [4, 5], even on operation.

Today, with the advance of imaging, the diagnosis of UD depends on the imaging more and more. US, CT, MRI, VCUG and CT-VCUG [4, 5] have been used as modalities for diagnosis of UD. None of them has ideal sensitivity and specificity, and no one can be considered as the golden diagnostic standard. Isolated studies have been tried to evaluate the diagnostic value of CT in female UD [6, 7].

In this article, we investigated the effectiveness of 640-MSCT in the diagnosis of female UD as a preoperative imaging.

## Materials and patients

Following the approval of our institutional review board, we performed 640-MSCT for 16 female patients with symptoms and physical examination suggestive of UD from August 2010 to March 2016. We reviewed the medical records of these 16 suggestive patients, including presenting symptoms, medical history, physical examination, cystourethroscopy, radiological imaging (especially 640-MSCT) and urinalysis preoperatively. We also analyzed intra-operative findings about the location and number of diverticular ostia. All patients were followed up for at least 3 months after surgery, and the follow-up results were also reviewed.

To evaluate the sensitivity and specificity of 640-MSCT on diagnosing female UD, we use 640-MSCT scanner to scan the patients’ pelvis to ascertain the existence of the UDs and to figure out their locations, sizes, ostia and shapes.

Images from 640-MSCT scanning were 3D and 4D reconstructed and reviewed by a radiologist, who was blinded to the surgical findings. The UD ostium was a tunnel between the urethra and UD, which communicated both. The existence of UD ostia was to determine whether the tunnel was visualized by the presence of contrast media in either 640-MSCT scanning image or 3D or 4D image. Its direction was defined as the ventral side clock position on the axial image. The length from the bladder neck to the ostia was estimated with 3D or 4D image. The shape of the diverticulum was evaluated and defined as pouch, U-shape and circumferential shape. The UDs that were round and located laterally or beneath the urethra were defined as pouch, that were around the urethra partially were defined as U-shape and that were around the urethra almost completely were defined as circumferential shape.

640-MSCT was performed with a Toshiba Aquilion ONE TSX-301A 640 Multislice volumetric CT. Our protocol was that the bladder was filled with 300 mL of contrast medium before scanning, and then, a 640-Multislice CT was performed for scanning the pelvis (from the top of the bladder to the inferior margin of the symphysis pubis) in the supine position. Thin-section spiral scanning was finished under the following conditions: dynamic volume acquisition CT (DVCT) pattern, 0.5 mm slice thickness, 160 mm Z axial detector width, 0.5 mm slice thickness for reconstruction, 512 × 512 matrix, 80 kV, and 5 s scanning time per series (total scanning time of 90 s), automatic tube current modulation, frame speed 0.35 s/lap, 0.25-mm layer spacing, open 3D applications of adaptive iterative dose reduction (AIDR). Following these, we obtained the image of the bladder’s maximum capacity. Then, the patients were instructed through microphone to void to get voiding and post-voiding images. To make sure of capturing the image during the whole voiding, 5 s were required for each scan. Thin-section images (0.5 mm) were used to generate 3D reformatted images. Continuous replay of these 3D images produced four-dimensional moving images (4D images).

Cystourethroscopy was performed on 10 patients under general anesthesia at the time of surgery by the same surgeon before the diverticulectomy. The UD ostia were closed horizontally by layered interrupted suture with absorbable sutures over a 14F or 16F Foley catheter.

The preoperative 640-MSCT images and 3D, 4D images about UD ostia were compared with operative, cystourethroscopic and other findings.

We did not perform statistical analysis because of the small number of patients.

## Results

From August 2010 to March 2016, we identified 16 women with UDs, which were compared with the surgical findings and other examination results. The mean age of patients was 38.8 years (range 26–53) at the time of diverticulectomy. The mean duration of symptoms presentation was 32.7 months (range 8–60). All patients (100%) suffered from dysuria, which was the most common symptom. Thirteen patients (81%) had dyspareunia. Seven patients (44%) were disturbed by post-void dribbling. Four patients (25%) had the histories of recurrent urinary tract infections. Pus discharge was observed in 2 patients (13%). Palpable anterior vaginal masses were found in 16 patients (100%). Four patients (25%) had purulent discharge extruded from the urethral meatus when the UD was compressed. Patients’ characteristics are described in Table 1

**Table 1:** Characteristics of 16 UD patients

Age (years)	Chief complaint	Shape of UD	Location of ostium (o’clock)		Num. of ostium		Flow-up	
			640-CT	Surg	640-CT	Surg	Duration (month)	Recurrence
26	Dysuria, dyspareunia, dribbling	Pouch	6	6	1	1	26	–
29	Dysuria, dyspareunia	Pouch	5	5	1	1	30	–
39	Dysuria, dyspareunia	Pouch	7	7	1	1	38	–
38	Dysuria, UTI, pus	U-shape	5	5	1	1	29	–
27	Dysuria, dyspareunia	Pouch	7	7	1	1	10	–
45	Dysuria, dyspareunia dribbling, pus	U-shape	5,7	5,7	2	2	8	–
43	Dysuria, UTI	Pouch	7	7	1	1	20	–
53	Dysuria, dyspareunia, dribbling, UTI, pus	Circumferential	5,6,7	5,6,7	3	3	33	+^a^
49	Dysuria, dyspareunia	Pouch	6	6	1	1	36	–
36	Dysuria, dyspareunia, dribbling	Pouch	7	7	1	1	48	–
37	Dysuria, dyspareunia	Pouch	5	5	1	1	25	–
32	Dysuria, dyspareunia	U-shape	7	7	1	1	50	–
39	Dysuria, dyspareunia, dribbling, UTI, pus	Pouch	5,7	5,7	2	2	60	+^b^
41	Dysuria, dyspareunia	Pouch	5	5	1	1	58	–
45	Dysuria, dribbling	Pouch	5	5	1	1	47	–
42	Dysuria, dyspareunia, dribbling	U-shape	7	7	1	1	6	–

*UD* urethral diverticula

^a^The second urethral diverticulectomy was done at post-op 4 months and cured

^b^The second urethral diverticulectomy was done at post-op 11 months and cured

**Fig. 1:**
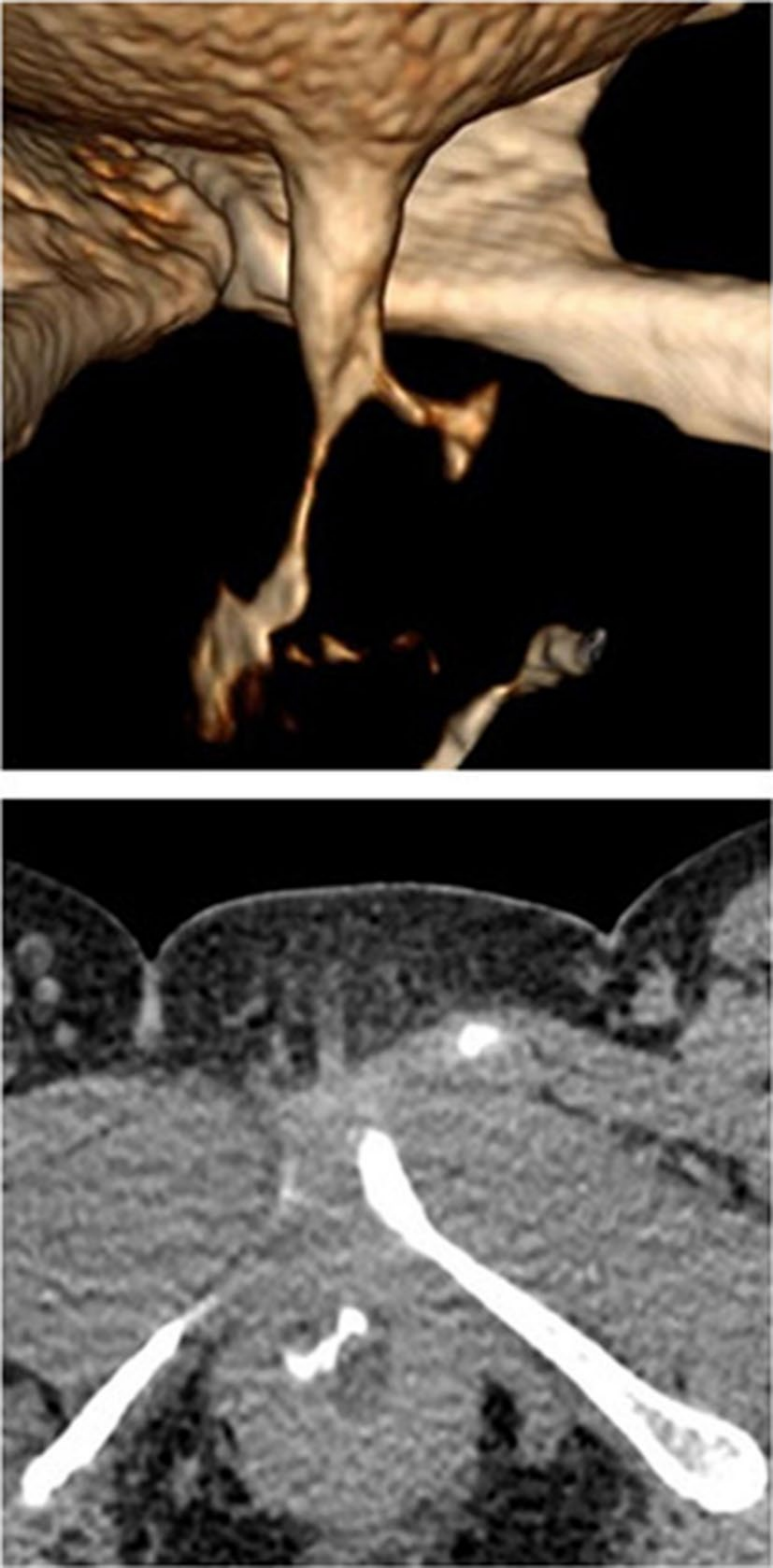
3D image of the beginning of voiding phase showed a pouch-shaped UD and the ostium (located at 5 o’clock); the ostium is thin but clear

**Fig. 2:**
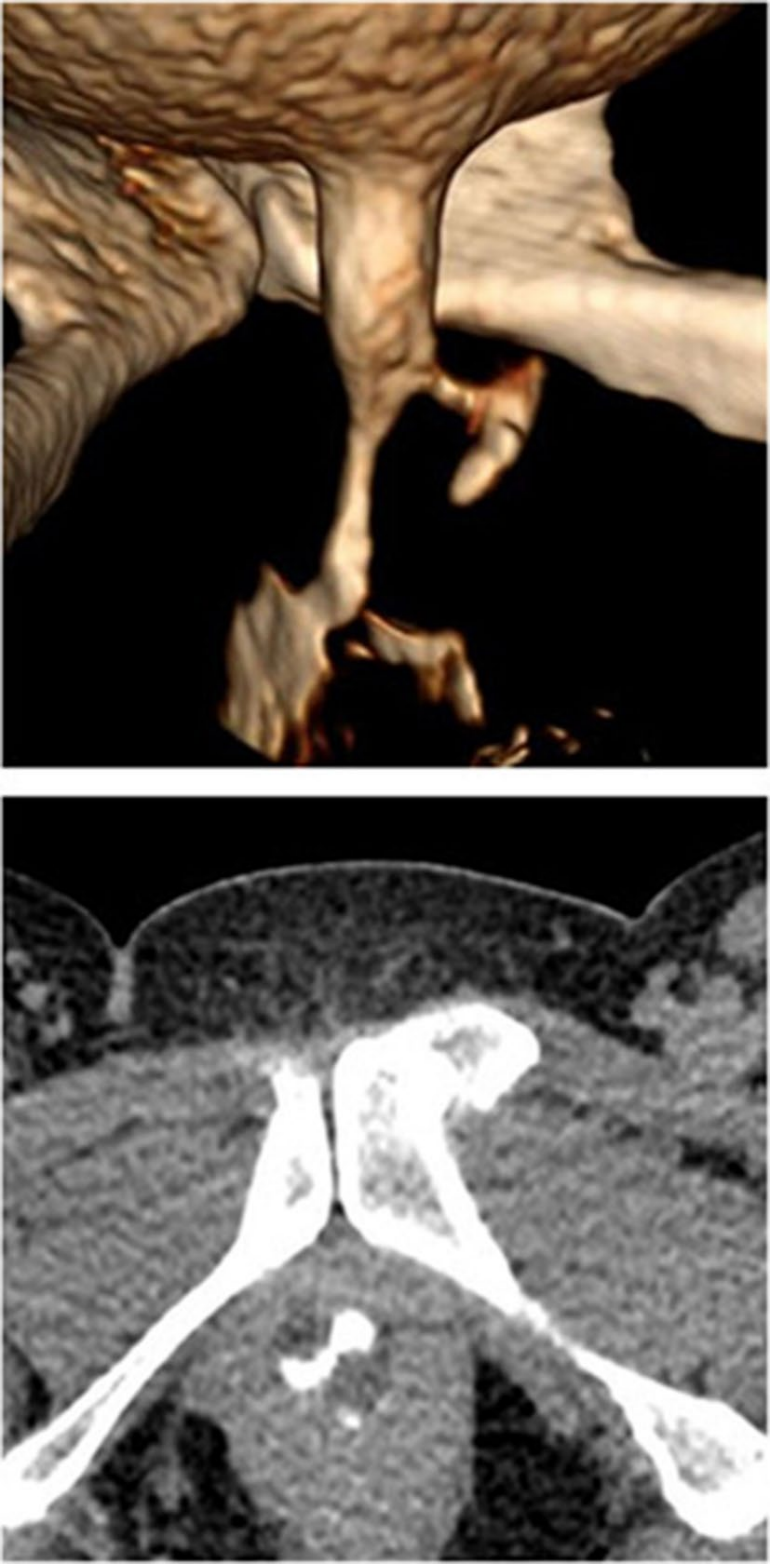
3D image of middle voiding phase showed a pouch-shaped UD and the ostium (located at 5 o’clock); the ostium is thick and clear

**Fig. 3:**
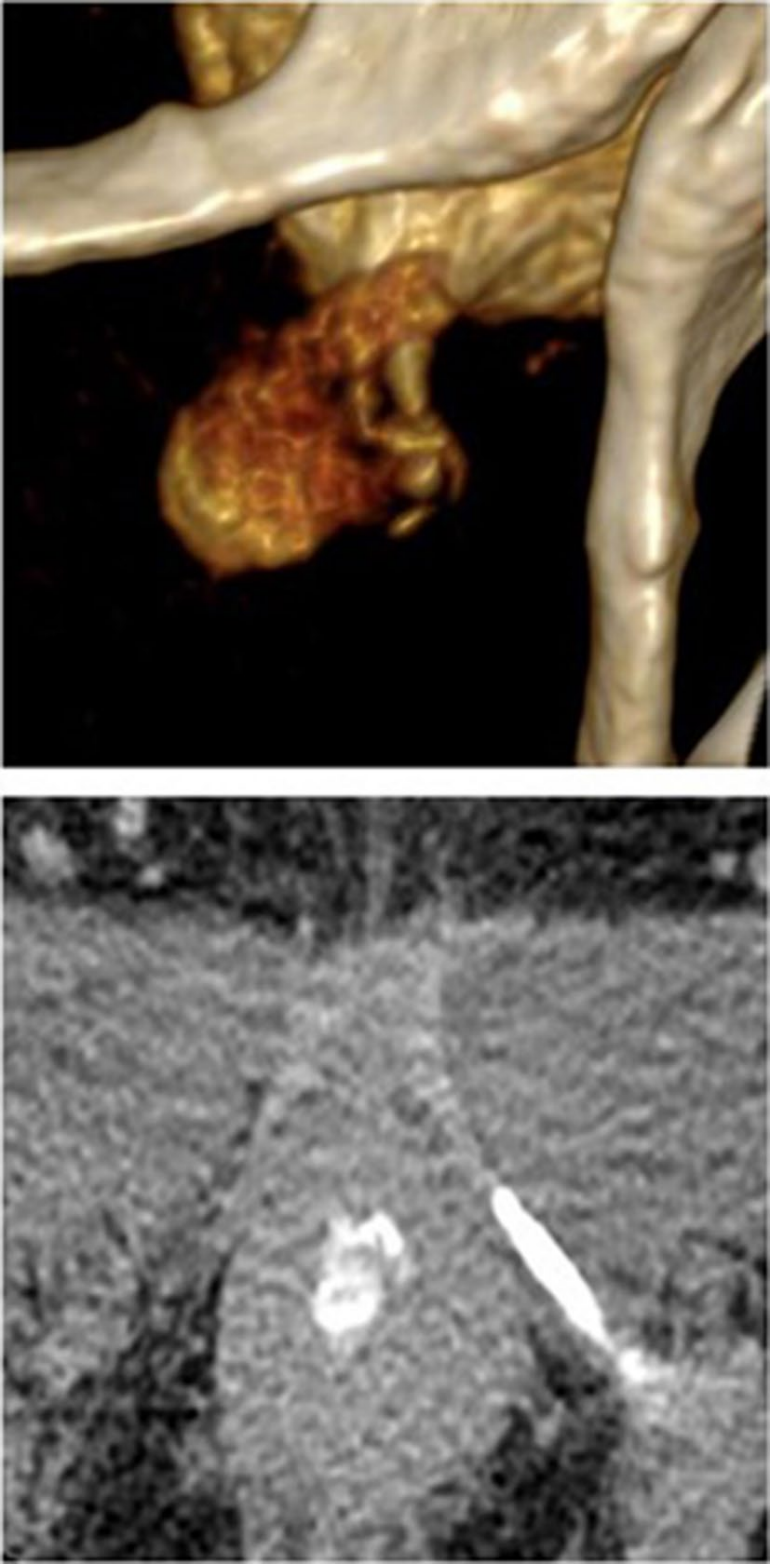
3D image of middle voiding phase showed a circumferential shape UD and the ostia (located at 5, 7 o’clock); the ostia is thick and clear

**Fig. 4:**
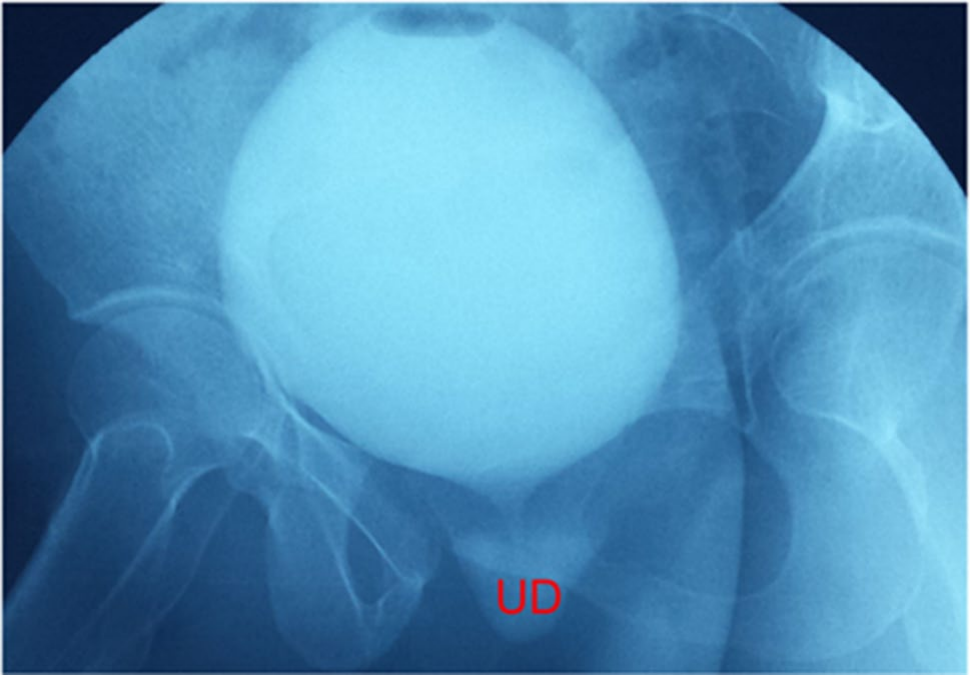
Voiding cystourethrogram of the same patient as Fig. 3. The UD could be seen, but the ostia could not be identified. *UD* urethral diverticulum

UDs characteristics are described in Table 2. Eleven patients (68.8%) had the diverticula of shaped pouch (Figs. 1 and 2). U-shaped diverticulum (not showed) was observed in four patients (25.0%) and circumferential shape UD (Fig. 3) existed in 1 patient (6.2%). In 16 patients (100%), the ostia could be identified clearly on 640-MSCT-3D or 4D image. At the beginning of voiding, the ostium is tinny and the diverticulum is small (Fig. 1), at the mid-voiding, the ostium is bulky and the diverticulum is larger (Fig. 2), and then before the end of voiding, the ostium become tinny again. We have done cystourethroscopy in 10 patients out of which four patients (40%) could be detected with ostia. Urethrography was done in eight patients, and none was identified the ostia, though the diverticulum could be seen (Fig. 4), especially the ostia which located between 4 and 8 o’clock direction, mainly between 5 and 7 (13 patients, 81.3%) o’clock position. Eight patients’ ostia (50%) located in 5 o’clock (Figs. 1 and 2), 5 patients’ (31.3%) located in 7 o’clock, one patient’s ostia (6.3%) located in 5 and 6 o’clock, one patient’s ostia (6.3%) located in 5 and 7 o’clock (Fig. 3), the other one’s (6.3%) located in 5, 6 and 7 o’clock. About the number of ostium, one patient (6.3%) had 3 ostia, located in 5, 6 and 7 o’clock and two ostia in 3 patients (12.5%; Fig. 3). One ostium was identified in 12 patients (81.3%). From the bladder neck to the ostium, the mean distance was 22.5 mm (range 15–29 mm). All the presentation of the UDs was confirmed surgically.

**Table 2:** UDs characteristics of sixteen patients

Age (years)	Shape of UD	Location of ostium (o’clock)				Num. of ostium			
		640-CT	Csto-	Urethra-	Surg	640-CT	Csto-	Urethra-	Surg
26	Pouch	6	Fail	–	6	1	Fail	–	1
29	Pouch	5	–	–	5	1	–	–	1
39	Pouch	7	7	–	7	1	1	–	1
38	U-shape	5	Fail	Fail	5	1	Fail	Fail	1
27	Pouch	7	–	–	7	1	–	–	1
45	U-shape	5,7	Fail	5,7	5,7	2	Fail	2	2
43	Pouch	7	–	Fail	7	1	–	Fail	1
53	Circumferential	5,6,7	6.7	7	5,6,7	3	2	1	3
49	Pouch	6	–	–	6	1	–	–	1
36	Pouch	7	Fail	–	7	1	Fail	–	1
37	Pouch	5	–	fail	5	1	–	Fail	1
32	U-shape	7	Fail	Fail	7	1	Fail	Fail	1
39	Pouch	5,7	5,7	5	5,7	2	2	1	2
41	Pouch	5	5	–	5	1	1	–	1
45	Pouch	5	Fail	Fail	5	1	Fail	Fail	1
42	U-shape	7	–	–	7	1	–	–	1

*UD* urethral diverticula

Diverticulectomy and closure of urethral defect were performed transvaginally on all sixteen patients. Fourteen patients (87.5%) were cured on first surgery, two were failed due to vaginal infection, and these two patients were cured by the following operation 4 and 11 months after the first surgery. No complications were found in 16 patients.

## Discussion

The incidence of female UD is increasing during the past 3 decades [8]. However, to diagnose the UD accurately, especially the ostium’s number and location, is still a challenging clinical problem. Diagnosis of UD is often delayed, often misdiagnosed for more common vulvovaginal cysts [3, 9]. In our cohort, none was diagnosed correctly and timely the first time patient went to see a doctor. One of the patients (53 years old) was diagnosed as vaginal cyst and underwent cystectomy by gynecologists. Her symptom recurred the first day after the operation, and she came to our hospital 2 months later.

The symptoms of female UD may be mistaken for other pelvic diseases. We diagnosed a suspicious UD, combining the symptoms with vaginal examination, cystourethroscopy and radiological imaging. We did vaginal examination for sixteen patients, and all were positive (100%). In our opinion, a good vaginal examination is a reliable index for the diagnosis of UD. The typical clinical manifestations are dysuria, dyspareunia and dribbling, which were present only in about one-third of the cases [10]. In our study, all patients (100%) presented clinically with dysuria, 13 patients (81.3%) presented with dyspareunia, 7 patients (43.8%) presented with post-void dribbling, 4 patients (25.0%) presented with UTI and 4 patients (25.0%) presented with pus discharge from urethral orifice. These clinical manifestations prompted us to exclude UD from dysuria, dyspareunia, dribbling, or UTI patients.

In the study of Lee et al. [4], the sensitivity of cystourethroscopy was 71.4%, and in the study of Pathi et al. [11], the sensitivities, specificities, positive predictive values (PPVs) and negative predictive values (NPVs) of cystourethroscopy were 33, 100, 100 and 42%, respectively. Similar to the study of Pathi, the sensitivity of cystourethroscopy in our group was only 40% (4 for 10).

Past studies and our cohort confirmed that radiological imaging is necessary to identify the UD and UD’s ostia, which is important in view of surgical treatment. In our study, 8 patients underwent voiding cystourethrography and three (37.5%) had positive results, lower than the previous study, which has an overall accuracy of 85% [12]. The ostia could not be identified in voiding cystourethrography (Fig. 4).

To a limited extent, we can identify female urethral abnormalities by conventional contrast-enhanced CT. A female UD may be diagnosed at CT images as a cystic mass. With the development of multidetector CT, especially the rapid image acquisition and post-processing techniques, 3D reformatted CT images, even CT-assisted virtual endoscopy can be available for diagnosis of UD and other urethral diseases [13, 14]. Using new-generation CT scanner of 640-MSCT, we obtained images of a contrast agent-filled urethra during patient voiding in approximately 5 s. This has led us to find more detailed urethral structure, which made it possible to identify the UD ostia easily and clearly. The high resolution of 640-MSCT made it feasible to detect the small amount of contrasts passing through the ostia, even the narrow ones. With the help of post-processing techniques, we reformatted 3D and 4D images, and all patients (100%) were diagnosed accurately pre-operation. The ostia were identified clearly and accurately. The results were in agreement with the operative findings. PPV of 640-MSCT was 100% in diagnosis of female UD.

MRI is considered as the new gold standard in diverticulum diagnosis, because it can provide excellent soft tissue contrast and delineate the UD shape and has the sensitivity of 100% in diagnosis of periurethral lesions [12, 15, 16]. A recent study showed the sensitivity, specificity, PPV and NPV of MRI for diagnosis of UD to be 100, 83, 9, and 100%, respectively [11]. However, another study showed that 24.4% of UD patients, diagnosed by MRI, had some discrepancy between the operative findings and the images of MRI, which made the search for the ostia difficult [17]. Some studies also show that MRI does not have excellent sensitivity in detecting ostia [15, 18]. In our study, 640-MSCT have been performed for all patients with reformatted 3D and 4D images. Sixteen patients (100%) were diagnosed accurately and identified by operative findings, with no discrepancy between 640-MSCT 3D, 640-MSCT 4D and operative findings. Among these patients, 3D and 4D show the UD to be in a shape of pouch in 11(68.8%) (Figs. 1 and 2), shape of “U” in 4 (25.0%) and circumferential shape in 1 (6.3%; Fig. 3). 3D and 4D pictures are superior in viewing ostia. In our group, thirteen patients (81.3%) have 1 ostium (Figs. 1 and 2), 2 patients (12.5%) have 2 ostia (Fig. 3), and 1 patient (6.3%) has 3 ostia (not shown). The location of ostia could be identified clearly, with 8(50.0%) in 5 o’clock position (Figs. 1 and 2), 6 in 7 o’clock (37.5%), one (6.3%) patient’s ostia located at 5 and 6 o’clock, one (6.3%) patient’s ostia located at 5 and 7 o’clock (Fig. 3), the other one (6.3%) located in 5, 6 and 7 o’clock (not shown). The mean distance from the bladder neck to the ostium was 22.5 mm (range 15–29 mm). All the presentation of the UDs was confirmed surgically.

Comparing to other modalities (such as MRI), this modality has higher sensitivity and specificity in diagnosing female UD, especially with the tiny ostia. This modality can identify cases which could be ignored in MRI image because in this modality we can view images dynamically in 4D view. For this advantage, the UD’s number, shape and location of ostium can be showed more clearly and accurately in 3D and 4D images.

In our experience, this modality can diagnose female UD accurately and can precisely direct the surgeon to perform the surgical procedure. According to the results of 3D/4D images, the surgeon can confirm the UD’s location, shape, and number and excise the UD perfectly and avoid omission of UD in multiple UDs’ patients. A few past studies have shown that the horse-shoe (our U-shape) or circumferential shaped UD or previous pelvic surgery may likely fail or recur [15, 19, 20]. The present study included 4 U-shaped UDs and 1 circumferential shaped UD, and the ostia could identify in these 5 patients. According to the 3D/4D images, all patients were cured after initial surgery, except the patient with circumferential shaped UD, in whom the first operation failed (she had an operation before) and one patient with pouch-shaped UD (she had infection in vaginal). Both of them were cured after the second surgery. In short, this modality could be beneficial to the surgical planning of diverticulectomy, especially in the planning of U-shaped or circumferential shaped UD.

Though 640-MSCT-3D and 4D have several advantages in diagnosis of UD, 640-MSCT-3D and 4D have a few disadvantages. First of all, we must discern this imaging modality carries ionizing radiation, which could hurt patients. We modified the protocol to reduce the adverse effects. Second, if the UD is large or filled with preexisting fluids, the contrast media cannot fill the lumen of UD, which may be interfere with the diagnosis. However, if we inspected the 4D images, we could find a scanty amount of contrast media through ostium into UD. Finally, some patients had difficulty in voiding on the CT table. Unfortunately, the scanning is only completed after the patients void while on the CT table.

The present study had several limitations. We could not provide the sensitivity and specificity of 640-MSCT-3D and 4D for the diagnosis of UD, because this is a retrospective study. We also could not do statistical analysis because of the small number of patients, which made the study be a descriptive study. Lastly, the follow-up modality was not uniform as we followed up patients using 640-MSCT-3D and 4D or transvaginal US or vaginal examination combined with clinical symptoms at 3 and 12 months postoperatively to confirm the disappearance of UDs.

Though the limitations exist, we present a usefulness of 640-MST-3D and 4D for diagnosis of UD. The ostia of UDs could be identified with 640-MST-3D and 4D preoperatively, including the location, number and UD shape, in all patients. And the results were confirmed by operative findings. To a suspicious UD patients or a planned surgical treatment patient, the modality is useful and recommended.
